# Infrared Bilateral Polarity Ship Detection in Complex Maritime Scenarios

**DOI:** 10.3390/s24154906

**Published:** 2024-07-29

**Authors:** Dongming Lu, Longyin Teng, Jiangyun Tan, Mengke Wang, Zechen Tian, Guihua Wang

**Affiliations:** 1 School of Electronic and Optical Engineering, Nanjing University of Science and Technology, Nanjing 210094, China; 2 Jiangsu Key Laboratory of Spectral Imaging & Intelligent Sense, Nanjing University of Science and Technology, Nanjing 210094, China

**Keywords:** infrared ship detection, bilateral polarity target, multi-feature, complex sea background

## Abstract

In complex maritime scenarios where the grayscale polarity of ships is unknown, existing infrared ship detection methods may struggle to accurately detect ships among significant interference. To address this issue, this paper first proposes an infrared image smoothing method composed of Grayscale Morphological Reconstruction (GMR) and a Relative Total Variation (RTV). Additionally, a detection method considering the grayscale uniformity of ships and integrating shape and spatiotemporal features is established for detecting bright and dark ships in complex maritime scenarios. Initially, the input infrared images undergo opening (closing)-based GMR to preserve dark (bright) blobs with the opposite suppressed, followed by smoothing the image with the relative total variation model to reduce clutter and enhance the contrast of the ship. Subsequently, Maximally Stable Extremal Regions (MSER) are extracted from the smoothed image as candidate targets, and the results from the bright and dark channels are merged. Shape features are then utilized to eliminate clutter interference, yielding single-frame detection results. Finally, leveraging the stability of ships and the fluctuation of clutter, true targets are preserved through a multi-frame matching strategy. Experimental results demonstrate that the proposed method outperforms ITDBE, MRMF, and TFMSER in seven image sequences, achieving accurate and effective detection of both bright and dark polarity ship targets.

## 1. Introduction

In navigation, searching, and tracking tasks under marine environments, infrared target detection technology plays a crucial role due to its unique advantages, such as long detection range, and high concealment [1,2,3]. Infrared imaging systems can obtain distance and shape information by receiving thermal radiation [4], and then produce infrared images for different tasks through subsequent processing. However, constrained by detector performance, complex weather conditions, and the inherent fluctuations of the marine, infrared images typically have only a narrow grayscale range, with a low contrast and restricted signal-to-noise ratio at long imaging distances, which significantly increases the difficulty of target detection.

In infrared ship detection tasks, targets can be roughly divided into point targets or small targets (having area less than 9 × 9 pixels, isotropic), area targets (typically having certain shape and contour information, lacking texture, but having a relatively uniform grayscale distribution), and larger targets (larger area, with rich texture and contour information). In point target detection tasks, researchers have delved into the isotropic shape characteristics and strong contrast of point targets, developing a series of efficient and reliable methods such as curvature filtering [5,6] and Local Contrast Measure (LCM) [7,8]. For larger targets, which usually occupy a significant area in the image and have rich contour and texture details, they are easier to detect compared to point and area targets, but face the challenge of how to completely extract the entire ship.

Area target ships always appear as patches with uniform grayscale distribution and regular shapes in infrared images. If the grayscale of the ship is greater (smaller) than its local area, it is called a bright (dark) polarity target. The existing detection methods of the area target ship can be roughly divided into histogram-based methods, background modeling methods, feature-based methods, and deep learning-based methods. Histogram-based detection methods rely on the grayscale distribution of pixels in the whole image, dividing the image into foreground and background categories under certain criteria. For different scenarios, many researchers have developed various histogram transformation methods to adjust the grayscale distribution of images [4,9,10], enhance the contrast of targets, and then use methods such as the Otsu [11,12], the maximum entropy [13,14], and various improved forms for segmentation. In addition, clustering methods [15,16] have been introduced into infrared ship detection tasks to achieve complete extraction of larger targets. Mean shift was utilized to smooth infrared images, enhancing the contrast of ship targets [17,18]. However, these methods often have strict limitations on the grayscale distribution of the image and the target, making it difficult to determine a reasonable segmentation threshold in complex scenarios with unknown target grayscale polarity, interference similar to real targets, or irregular histogram distribution. Background modeling methods estimate the background in various ways to separate the target from the background. The Infrared Patch Image (IPI) model [19] is based on the assumption that the target is sparse with the background low-rank; thus, by dividing, re-organizing, decomposing, and reconstructing the image, small-sized targets in a stable background can be detected. Subsequently, researchers have improved the reconstruction and decomposition methods of sub-images to enhance the detection capability of the IPI model in complex scenarios [20,21]. Apart from this, researchers use Gaussian mixture models to model the background [22,23,24], achieving prediction and reproduction of dynamic backgrounds. These methods have certain robustness in scenarios with severe fluctuations but struggle to distinguish irregular fish scale reflections, sun-glint, and other interferences in complex scenarios.

In infrared images, ships often have one or more features that make them distinguishable from the background. By quantitatively analyzing these features and segmenting images according to certain criteria, feature analysis-based detection algorithms can be formed. Top-Hat filtering was first introduced into infrared ship detection tasks [25], using morphological filters to suppress clutter in the image while preserving bright blobs, achieving the detection of bright ships. On this basis, many researchers have improved morphological operations, such as using annular structural elements to enhance the detection capability for small-sized targets [26,27] or introducing multi-scale algorithms to achieve adaptive detection of targets of different sizes [28]. In addition, features such as contours [29,30] and gradients [31,32,33] have also been widely used in ship target detection, and many researchers consider combining multiple features [15,34] to enhance the robustness of algorithms in different scenarios. For dark ships, many researchers have conducted in-depth studies. Dong et al. [35] calculated saliency maps through an inverse Gaussian difference filter, making dark blobs outstanding in the saliency map, and then extracted potential ships in the image by segmenting the saliency map with an adaptive threshold. However, this method struggles to distinguish narrow dark bands on the sea surface or small-sized fish scale patterns with clear edges.

By using Grayscale Morphological Reconstruction operations to preserve and suppress bright or dark blobs in infrared images, Li et al. [36] achieved parallel detection of bright and dark ships. However, this method also faces difficulties in determining segmentation thresholds in complex scenarios with “significant” interference. To address the interference of island and reef backgrounds, Chen et al. [37] calculated the improved structural tensor of the multi-scale grayscale morphological reconstruction results of the original infrared image as a guide, merging the prominent regions in the Gaussian-filtered image to detect bright polarity ships of different sizes. However, the improved structural tensor proposed by this method struggles to distinguish fish scale pattern interference similar in size to ships. Ding et al. [4] proposed an improved histogram equalization combined with gradient information (MHEEF) to preprocess infrared images with backlit scenes, enhancing the contrast of dark ships, and then a dual-scale, dual-mode Local Contrast Measure (LCMDSM) was utilized to extract targets. The above methods can all be summarized as using single-frame image information to detect ships in single-frame images. Considering that ships, as man-made objects, have temporal and spatial stability in continuously captured image sequences, based on this feature, Wang et al. [31] proposed an improved wavelet transform to suppress the time-varying background clutter and simultaneously track stable ships by using pipeline filtering. Similarly, Li et al. [38] first extracted the Maximally Stable Extremal Regions (MSER) in the infrared image, then suppressed clutter through region matching between adjacent frames, and finally stable bright and dark polarity ships were detected at the same time. However, when the sea surface fluctuates violently and the size of the ship is small, it will be challenging to achieve stable matching between the MSER regions containing ship targets directly extracted from different frames, which may lead to frequent missed detections. Zhang et al. [39] developed a “detection-tracking-detection” method for detecting small-sized bright ships in infrared images, first extracting regions in single-frame images where targets may exist by using difference of Gaussian filtering and adaptive threshold segmentation, then suppressing interference through continuous frame matching. Apart from this, a re-detection method for potential missed targets was designed to further improve the robustness of this method.

As a trend in many research fields, deep learning-based methods are data-driven, that is, through data annotation, reward and punishment mechanisms, as well as iterative training, researchers enable neural networks to mine and refine deeper, more abstract features from a large amount of data and finally achieve efficient and accurate detection. Early on, such methods were mainly applied to infrared image target detection tasks with a space-based observer [40,41]. In 2018, Zhou et al. [42] proposed a one-stage network to learn features from multi-resolution infrared images, achieving reliable detection of ships in large infrared images. In 2022, Long et al. [43] introduced a visual attention mechanism into the YOLOv5 network architecture and introduced dilated convolution to enhance the receptive field, achieving the recognition of infrared ships against the background of a gentle sea surface with island reefs. By combining a manually designed feature extractor and deep learning methods, Yao et al. [44] designed a multi-dimensional information fusion network to accurately identify small-sized bright ships in infrared images. In 2023, Deng et al. [45] published an infrared ship rotating target detection algorithm, FMR-YOLO, in which a Weighted Feature Pyramid Network Based on Extended Convolution (DWFPN) was proposed with rotation detection technology introduced and achieved an average accuracy of up to 92.7%. Considering the complexity of the deep learning method and the difficulty of deployment on small devices, Gao et al. [46] proposed a lightweight model for detecting infrared ships by replacing the backbone of YOLOv5 with the Mobilev3, which greatly improved the computational efficiency and achieved the same detection performance as the YOLOv5m model while reducing the parameter size by 83%. In 2024, an improved detection model based on YOLOv5s to detect infrared ship targets in coastal areas with high ship density and significant target scale differences was proposed by Wang et al. [47], in which a feature fusion module was designed to enhance the feature fusion of the network, with SPD-Conv and Soft-NMS adopted to improve the detection accuracy of small targets in low-resolution images and deal with the missed detection in the case of dense occlusion. In addition to improving the design of the model, Wang et al. [48] introduced infrared multi-band fusion technology to improve detection accuracy with fewer parameters, achieving inference speeds close to 60 frames per second on embedded devices. Apart from this, many researchers [49,50,51] have been applying deep learning-based methods into ship detection tasks in infrared remote sensing images, continuously improving the model performance and detection effect. However, for deep learning-based methods, a large amount of training data are needed to ensure the reliability of the neural network. For example, in [43], researchers mentioned using 4079 out of 4533 infrared images for training and then testing the remaining images. On the one hand, publicly available infrared sea surface image datasets vary greatly and are limited in number. At the same time, manually annotating a large number of infrared images with poor contrast, missing target details, and a low signal-to-noise ratio requires a lot of manpower and resources, still posing challenges for deep learning-based methods [37].

In summary, we may summarize the current challenges in detecting infrared ships. First, most existing methods are designed for relatively simple scenarios with smooth seas, and usually a single grayscale polarity of the target is assumed, while in actual sea surface scenarios, the polarity of ship targets in infrared images is often unknown due to the variation in sea conditions, illumination, and detector positions, thus may result in missing detections. Second, in complex scenarios, there may exist interferences of different sizes, such as islands, artificial structures, bright and dark bands, fish scale patterns, and even clouds, which may be more prominent than real targets in the saliency map of various features. As a result, it may struggle to determine the segmentation parameters for methods such as adaptive threshold segmentation or Otsu to achieve balance between accuracy and completeness. Finally, in some scenarios, the temporal and spatial stability of the ships may not be fully utilized, and these features may provide some assistance for infrared ship detection tasks.

In this paper, we make the following assumptions regarding area target ships in maritime scenarios:

(1) Ship Polarity: Ships can exhibit either bright polarity or dark polarity. Specifically, their grayscale values are either relatively high (bright) or low (dark) than the local background.
(2) Uniform Grayscale Distribution: The grayscale distribution of ships is uniform across the infrared image sequences.
(3) Temporal and Spatial Stability: Ships demonstrate temporal and spatial stability in infrared image sequences. In other words, their grayscale distribution and shape remain nearly constant over time.

Addressing the issues above, this paper first proposes an infrared image smoothing method that combines GMR and RTV to suppress noises and enhance the contrast of ships. Subsequently, the Maximally Stable Extremal Regions in the image are extracted as candidate targets. Finally, shape features and spatiotemporal characteristics are integrated to discriminate between ships and interferences, achieving the detection of bright and dark ships in complex scenarios.

## 2. Materials and Methods

**Figure 1:**
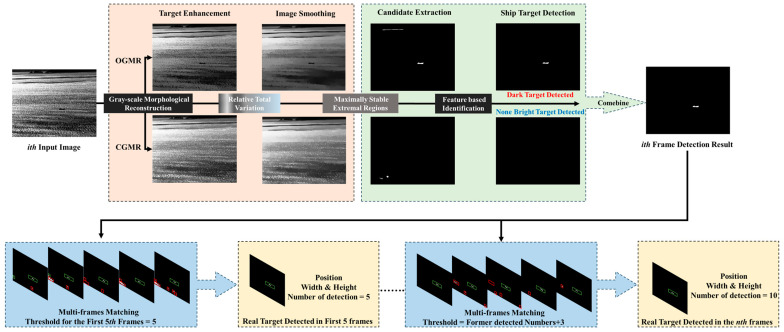
The framework of the proposed method.

The framework of the proposed method is illustrated in Figure 1, primarily consisting of image smoothing based on the GMR and RTV, candidate region extraction based on MSER and shape features, and multi-frame matching based on spatiotemporal characteristics. In this section, the principles of the GMR and RTV algorithms used for image smoothing and sea clutter suppression are introduced first. Subsequently, we extract MSER as the candidate targets from the smoothed images. Finally, spatiotemporal features are introduced to detect ships and suppress interferences.

### 2.1. Grayscale Morphological Reconstruction

Grayscale Morphological Reconstruction (GMR) is widely used as a powerful tool in image preprocessing and segmentation due to its excellent performance in image feature extraction and image restoration [36]. Taking the results of the opening (closing) operation of the original image as the constraint of iterative geodesic dilation (erosion), the GMR can be divided into opening-based (*OGMR*) and closing-based (*CGMR*), which can be used to extract the connected domain in the image with a uniform gray distribution that is darker (brighter) than the surrounding pixels, respectively. The definition of *OGMR* is as follows:

(1) $$OGMR_{I}\left(I_{open}\right)=\underset{k\geq 1}{\vee }gd^{k}(I_{open}),$$

(2) $$gd^{1}(I_{open})=\wedge \left[I_{open}⊕b,I\right],$$

(3) $$gd^{k}(I_{open})=gd^{1}\left(gd^{k-1}(I_{open})\right),$$

where *I_open_* is the opening result of the original image *I* and *k* and *b* represent the iterations and the structure element of the geodesic dilation *gd*, respectively. ∨ and ∧ represent the pixel-wise maximum operation and minimum operation, respectively. With *I* the mask image, geodesic dilation is iteratively executive until stability is reached according to Equation (3), in which the bright blobs have been suppressed while the dark blobs persevered with their contour unchanged.

Replace the marker image with *I_close_* and perform iterative geodesic erosion *ge^k^*; similarly, the definition of *CGMR* is as follows:

(4) $$CGMR_{I}\left(I_{close}\right)=\underset{k\geq 1}{\wedge }ge^{k}(I_{close}),$$

(5) $$ge^{1}(I_{close})=\vee \left[I_{close}⊙b,I\right],$$

(6) $$ge^{k}(I_{close})=ge^{1}\left(ge^{k-1}(I_{close})\right),$$

**Figure 2:**
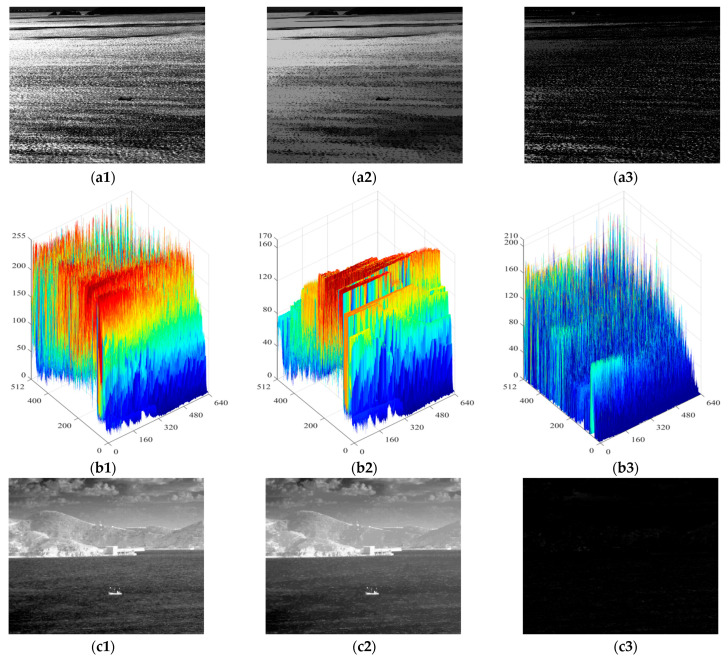

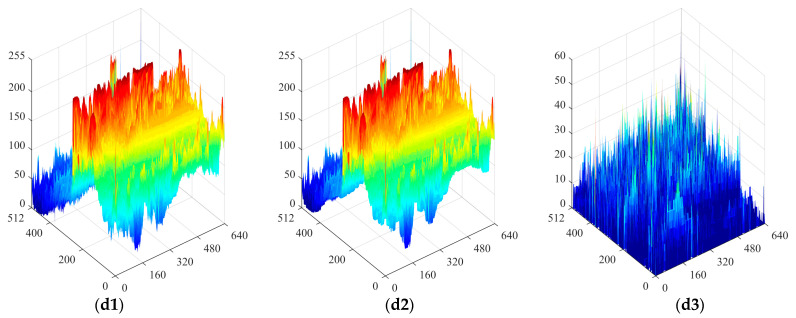
The input images and the results of *OGMR*(*CGMR*). (**a1**–**a3**) and (**b1**–**b3**) are the input dark target image, the result of *OGMR* processing, *CGMR* processing, and the corresponding three-dimensional view of gray distribution, respectively. (**c1**–**c3**) and (**d1**–**d3**) are the input bright target image, *CGMR* processed images, *OGMR* processed images, and the corresponding 3D views of gray distribution, respectively.

In contrast, the *CGMR* operation suppresses dark blobs while preserving bright blobs with almost unchanged contours. In this paper, a disc-shaped structural element with a radius of 15 pixels is employed in the opening and closing operation to obtain the marker image, followed by iterative geodesic dilation/erosion using a 3 × 3 pixels square structural element, *b*. The results, as shown in Figure 2, demonstrate that *OGMR* (*CGMR*) helps preserve dark (bright) polarity targets while suppressing the opposite. Given that ship targets in infrared images often manifest as bright or dark blobs, and with the intention of achieving both bright and dark ships in single-frame detection, this paper applies *OGMR* and *CGMR* separately to the input images to extract potential bright and dark polarity targets concurrently. However, in complex maritime scenarios, under the combined effects of sea wind, illumination, and other factors, there is a possibility of forming interference regions similar to real ships on one hand. And on the other hand, the uniformity of the ship’s grayscale distribution may be compromised, affecting the regularity of the contours. In such cases, methods like the adaptive threshold segmentation mentioned in [36] or the improved structural tensor used in [37] may struggle to distinguish between real ships and a large number of interferences. To address this issue, this paper introduces Relative Total Variation into the ship target detection process, aiming to effectively suppress sea clutter in infrared images.

### 2.2. Relative Total Variation

Relative Total Variation (RTV) is an algorithm proposed by Li et al. [52], which can achieve effectively smoothing textures within the input image while preserving and extracting the structure. The fundamental idea of RTV is to distinguish between larger-scale structures and smaller-scale textures by using the ratio of the sum of the weighted absolute gradient values (referred to as windowed total variations) to the absolute value of the sum of the weighted gradients (referred to as windowed inherent variations) within a sliding window. This ratio, that is, the relative total variation, is then used as a penalty term in the objective function. The definition of RTV is as follows:

(7) $$\arg\underset{S}{\min}{\sum_{p}\left(S_{p}-I_{p}\right)}^{2}+\lambda \cdot \left(\frac{D_{x}(p)}{L_{x}(p)+\varepsilon }+\frac{D_{y}(p)}{L_{y}(p)+\varepsilon }\right),$$

(8) $$D_{x}(p)=\sum_{q\in N(p)}g_{p,q}\cdot \left|{\left(\partial_{x}S\right)}_{q}\right|,D_{y}(p)=\sum_{q\in N(p)}g_{p,q}\cdot \left|{\left(\partial_{y}S\right)}_{q}\right|,$$

(9) $$L_{x}(p)=\left|\sum_{q\in N(p)}g_{p,q}\cdot {\left(\partial_{x}S\right)}_{q}\right|,L_{y}(p)=\left|\sum_{q\in N(p)}g_{p,q}\cdot {\left(\partial_{y}S\right)}_{q}\right|,$$

where *S* and *I* are the input and output-smoothed image, respectively. *q* is the index of the pixels in the sliding window with *p* as the center. *λ* is the parameter that controls the degree of smoothing, and *ε* a constant to prevent the denominator from being 0. *D_x_*(*p*), *D_y_*(*p*), and *L_x_*(*p*), *L_y_*(*p*) represent the windowed total variations and the windowed inherent variations of pixel *p* along *x* and *y* directions. The sliding window *g_p_*_,*q*_ is in fact a Gaussian filter, with its standard deviation *σ* corresponding to the maximum scale of the unfiltered texture.

**Figure 3:**
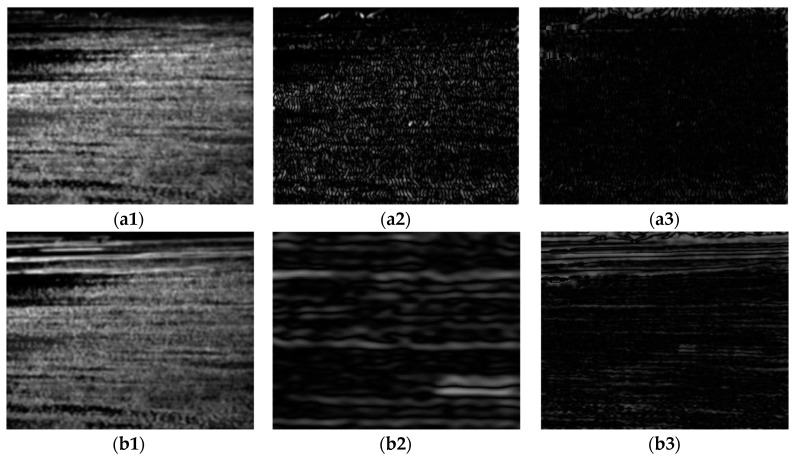
Taking the dark target image as an example, (**a1**–**a3**) and (**b1**–**b3**) are the results of windowed total variation, windowed inherent variation, and inverse relative total variation of the original infrared image (Figure 2(a1)) and the result of *OGMR* processing, respectively.

**Figure 4:**
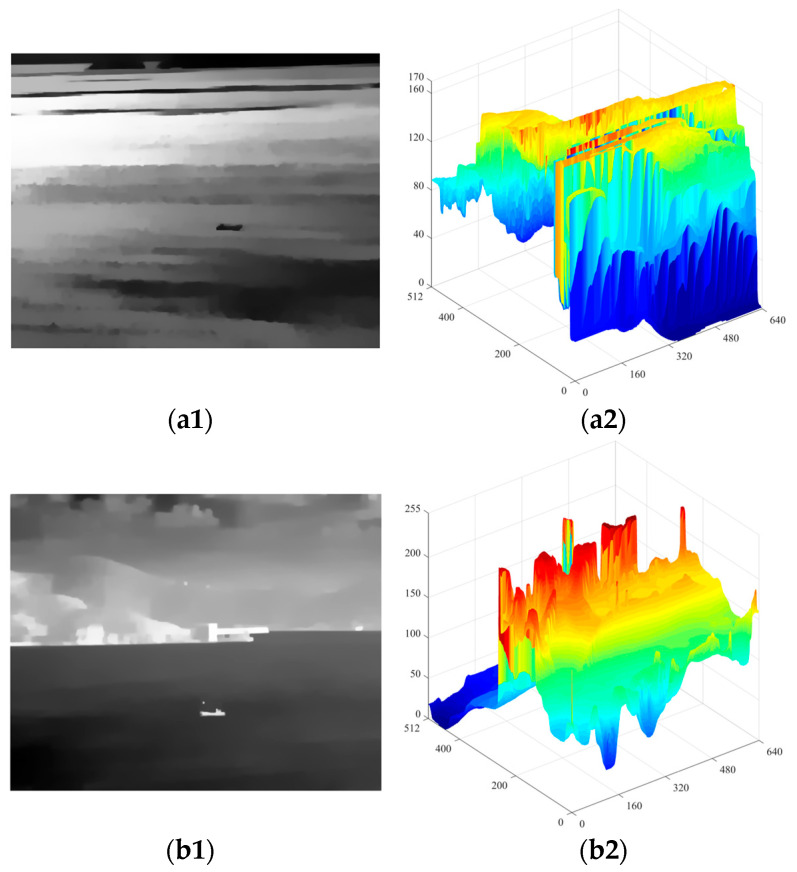
The smoothed results of the image with a dark ship (Figure 2(a2)) and the image with a bright ship (Figure 2(c2)) with RTV. (**a1**,**a2**) are the grayscale distribution and the three-dimensional view of the dark ship image, respectively. (**b1**,**b2**) are the grayscale distribution and the three-dimensional view of the bright ship image, respectively.

By transforming the original objective function into linear equations, the output image *S* can be obtained by iteratively solving those equations. In this paper, we set the parameters *λ*, *σ*, and the number of iterations *t* to 0.015, 3, and 3, respectively, in most cases. Figure 3 shows the RTV results obtained directly from the original image and the image performed by *OGMR*. It can be observed that in the results of windowed total variation, smaller-scale textures such as fish-scale patterns are quite prominent, whereas in the results of windowed inherent variation, larger-scale structures with consistent gradient orientations within the sliding window, such as ships, edges of reefs, and boundaries of bright and dark bands, are more notable. In the results of the reciprocal relative total variation, by taking the ratio of the former two, the differences between small-scale clutter and large-scale structures, that is, the differences between textures and structures, are further amplified. The more prominent parts reflect large-scale structures such as reefs, dark bands, and ships. Based on the aforementioned analysis, using the relative total variation model for iterative processing of infrared images can effectively suppress small-scale clutter in the background while enhancing the grayscale uniformity across different regions of the image. After performing *OGMR* (*CGMR*), connected domains of a single polarity are preserved, while those of the opposite polarity are suppressed, and the magnitude and directional characteristics of image gradient changes are restrained to some extent. Subsequent smoothing with the RTV model, on this basis, can achieve the desired smoothing effect with fewer iterations. At the same time, small-scale connected domains of the same polarity preserved in the reconstructed image are suppressed after smoothing, reducing the potential interference. The smoothed infrared image and its grayscale distribution are shown in Figure 4. This paper processes the input infrared images with two types of GMR as the input for detecting bright and dark ships, followed by RTV smoothing as the input for candidate extraction.

**Figure 5:**
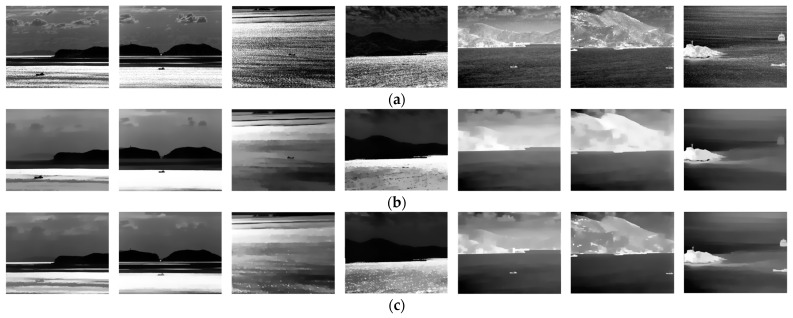
The smoothed results of images in different scenarios. (**a**) consists of the original input infrared images. (**b**,**c**) are the smoothed results of *OGMR* and *CGMR*, respectively.

Apart from this, the smoothed results of infrared images in diverse scenarios are displayed in Figure 5, and the numerical indicators of the corresponding image sequences are also displayed in Table 1. Here, the input images were set as references to evaluate the effect of the smoothing method in this paper, and we adopted the peak signal-to-noise ratio (*PSNR*) and the mean Structural SIMilarity (*SSIM*) index [53] as numerical indicators. The definitions of the *PSNR* and mean *SSIM* are as follows:

(10) $$PSNR=10\cdot \log_{10}\left(\frac{255^{2}}{MSE}\right),$$

(11) $$mSSIM=\frac{1}{M\times N}\sum_{i=1}^{M}\sum_{j=1}^{N}SSIM(x_{i},y_{j}),$$

where *MSE* is the mean-square error between input images and smoothed images, and *SSIM* is the Structural SIMilarity index of image patches calculated by using a Gaussian window with a size of 11 × 11 pixels and a standard deviation of 1.5, in which *M* × *N* is the number of image patches. As shown in Figure 5 and Table 1, the proposed smoothing method can effectively suppress interferences like the fish scale pattern and improve the signal-to-noise ratio, while inevitably resulting in the loss of image structure information.

### 2.3. Maximum Stable Extreme Region

In the results of the abovementioned smoothed images, the contrast between the ship and its local background is further enhanced, resulting in a more uniform gray distribution of whole images. Given the grayscale uniformity inherent to the ships, the Maximally Stable Extreme Region (MSER) algorithm can be adept at extracting the isolating connected domains ranging between [81, 1500] pixels as potential targets. The principle of extracting the MSER is to binarize the image by increasing (decreasing) the segmentation threshold step by step, and find out regions with minimal area variation, that is, the so-called maximum stable extreme region. Regions with more uniform grayscale distribution are more likely to maintain stability in area size under different thresholds, indicating a lower area change rate. The area change rate is defined as follows:

(12) $$p(i)=\frac{\left|Q_{j+\Delta }-Q_{j-\Delta }\right|}{Q_{j}},$$

in which *Q*_1_, *Q*_2_, …, *Q*_j_, … represent a series of nested regions obtained as the threshold increases from 0 to 255 (or decreases from 255 to 0) in steps of Δ; |∙| is the area of a region, that is, the number of pixels. If one particular region *Q*(*j*) has an area change rate *ρ*(*j*) less than a predefined threshold *Tρ*, it is considered maximally stable. In this paper, the area change rate threshold *Tρ* is consistently maintained at 0.25. Research [38] suggests that typically, a smaller step size Δ results in a greater number of MSERs extracted per frame, while a larger step size will only extract those with a higher uniform grayscale distribution. On the one hand, based on the characteristic of ships having a more uniform grayscale distribution compared to their background, the range of step size Δ can be appropriately amplified to reduce the number of clutter regions mistakenly extracted. On the other hand, in complex maritime scenarios, there may exist situations where ships have rather low contrast against their background, in which smaller ships can easily blend into the background. If a smaller step size Δ is chosen in such scenarios, large areas containing ships are more likely to be extracted rather than the ships themselves, leading to potential misjudgments in subsequent analysis.

**Figure 6:**
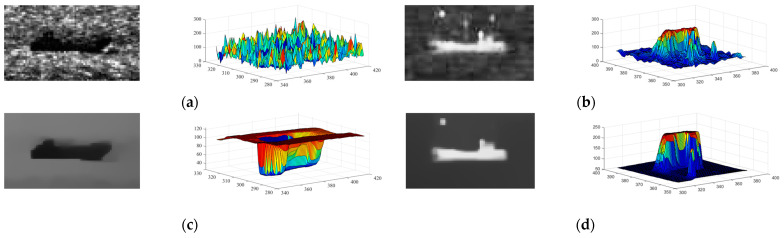
Grayscale distribution of dark (light) ships and surroundings before and after smoothing. (**a**,**c**) are the grayscale distribution and corresponding 3D views of the dark ship and local background in the original infrared image and smoothed result by *OGMR* and RTV, respectively. (**b**,**d**) are the grayscale distribution and corresponding 3D views of the bright ship and local background in the original infrared image and smoothed result by *CGMR* and RTV, respectively.

Figure 6 illustrates the pixel grayscale distribution within the local background of the ship before and post-smoothing. The results indicate that after smoothing, the grayscale within the actual ship target zone is markedly uniform, exhibiting a significant contrast with the background. This contrast facilitates the selection of a larger step size Δ, which not only accurately extracts the ship target but also minimizes the extraction of extraneous clutter. However, it has to be mentioned that while the smoothing process effectively suppresses smaller clutters, it may inadvertently homogenize certain regions of the sea surface background, previously contained clutters, before smoothing, rendering them as potential candidate targets. In this paper, the outputs from the two aforementioned channels are independently processed and subsequently combined to form the final candidate targets. Taking into account the relative spatiotemporal stability of the ship against the dynamic sea surface background and the inherent variability of the background clutter, we employ both shape features and a pipe filtering approach to further exploit the difference between the ship and the clutter. Interference is screened out during single-frame detection with a manually designed shape feature range, while concurrently eliminate false positives through the aggregation of single-frame detection results and inter-frame matching, thereby ensuring the retention of genuine ships.

### 2.4. Shape Feature-Based Target Extraction

Following the smoothing and extraction processes above, candidate targets are separated from the local background and rendered into binary form to establish distinct candidate target regions. Because their shape and contour features are largely preserved, they provide a reliable foundation for verifying real ships and interferences. Shape features play a pivotal role in infrared ship detection tasks, offering an effective means to identify ships amidst sea clutter, islands, reefs, clouds, and other forms of interference. Typically, ship targets exhibit a narrow form, with the upper portion being smaller than the lower, coupled with a relatively uniform contour. Leveraging these shape features, metrics such as aspect ratio, rectangularity, compactness, and the ratio of the upper to lower area are employed as shape features to authenticate targets. The definitions of these features are as follows:

(13) $$Ratio_{width\&height}=\frac{width}{height},$$

(14) $$Compactness=\frac{{\left(perimeter\right)}^{2}}{area},$$

(15) $$Rectangularity=\frac{area}{area_{Rec}},$$

(16) $$Ratio_{up\&down}=\frac{area_{up}}{area_{down}},$$

where *width* and *height* are the size of the minimum external rectangular box of the candidate target. *perimeter* and *area* are the perimeter and area of the candidate target, respectively. *area_Rec_* is the area of the minimum external rectangular box, and *area_up_* and *area_down_* are the areas of the upper and lower parts of the vertically equally divided bounding box.

The aspect ratio serves as a criterion to filter out interferences that manifest as excessively narrow strips. Compactness and rectangularity are utilized to eliminate clutter with relatively irregular contours. Furthermore, this study focuses on ships with an area ranging from 81 to 1500 pixels. Consequently, an area threshold is established to eliminate any interference falling outside this range. We selected 264 different ships from the VAIS dataset [54], 200 ships from the IRay dataset, and 100 infrared ship images from the self-collection dataset for analysis and summarization and obtained the statistical parameters of shape features as presented in Table 2. The IRay dataset is an open-source infrared maritime ship dataset provided by Yantai Raytron Technology Co., Ltd. (Yantai, China), which consists of thousands of infrared images containing various types of ships and can be accessed at http://openai.iraytek.com/apply/Sea_shipping.html/ (accessed on 9 June 2021). Additionally, the self-collection dataset in this paper comprises image sequences of infrared ships with different polarities captured from multiple coastal viewpoints.

**Figure 7:**
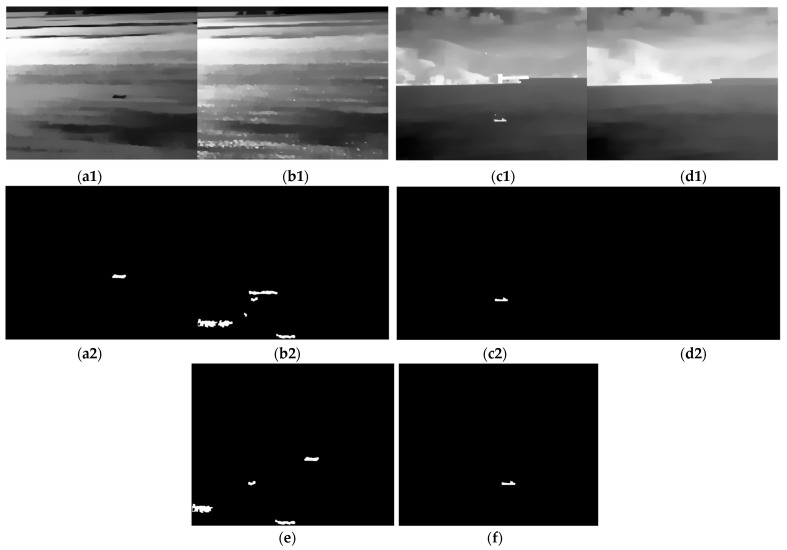
The results of the MSER extracted from the infrared image after smoothing and the results of the screening by shape features, with the step size Δ = 3.5 in the dark ship image and the step Δ = 8 in the bright ship image. (**a1**,**a2**) and (**b1**,**b2**) are the results of the dark ship image processed and smoothed by *OGMR* (*CGMR*) and the extracted candidate targets, respectively. (**c1**,**c2**) and (**d1**,**d2**) are the candidate targets extracted after *CGMR* (*OGMR*) processing and smoothing, respectively. (**e**,**f**) are the results of the merging of two channels of dark ship image and bright ship image, respectively.

Figure 7 illustrates the results of further screening candidate targets by employing the previously defined shape feature parameters. These shape features can be used to eliminate a portion of the interference; however, in complex environments, certain clutter with intricate shapes may still conform to the specified criteria. Therefore, the differences in spatiotemporal stability between targets and interference are introduced to make further distinctions.

### 2.5. Spatiotemporal Stability-Based Multi-Frame Matching Strategy

Apart from the utilization of shape features, many researchers have developed multi-frame detection methods for small targets, capitalizing on target stability, as referenced in [6,55]. The constancy of area targets within image sequences, particularly for larger ships, has been thoroughly investigated as well [31,39], culminating in the development of various algorithms. Pipeline filtering, a “tracking before detection” (TBD) strategy, is widely employed in infrared image target detection tasks. This technique begins with each prospective target in the reference frame, determining an optimal pipeline radius—corresponding to the detection scope in adjacent frames—based on the target’s size. Concurrently, an appropriate pipeline length threshold is established, representing the requisite number of detections and matching occurrences for a potential target within the image sequence. A potential target is deemed to be a real target only if its detection frequency surpasses the threshold; otherwise, it is classified as a false target.

**Figure 8:**
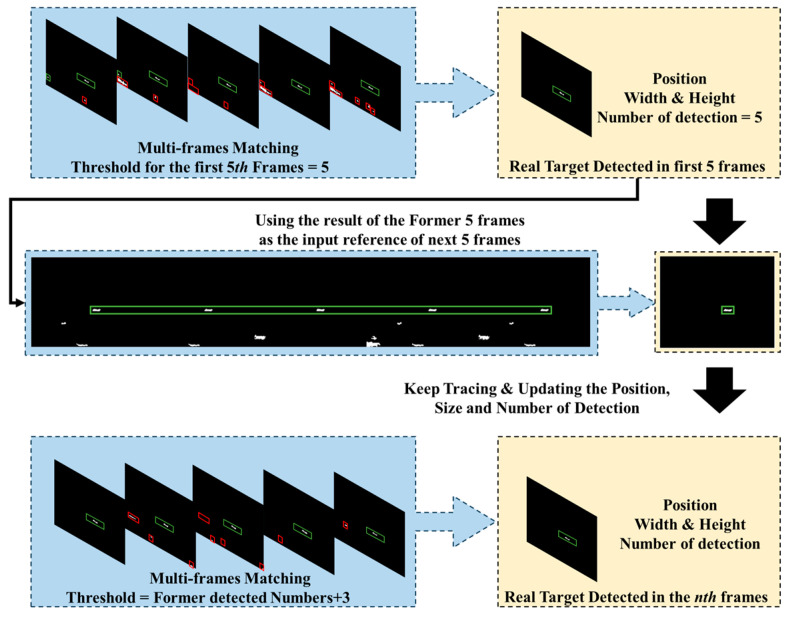
The framework of multi-frame matching.

Inspired by the idea of pipeline filtering, this paper designs a simple multi-frame detection approach to further utilize the spatiotemporal stability of ship targets. This method significantly diminishes false alarm interference by accumulating the results of single-frame detections across image sequences. The conceptual framework of this method is depicted in the subsequent Figure 8, utilizing the outcomes of shape feature analysis as inputs and employing a sequence of 5 adjacent frames for multi-frame detection and matching. The process commences with the designation of the initial frame from the five-frame sequence as the reference frame, labeled as *i* = 1. The coordinates, width, and height of all potential targets *C_i,_*_1_, …, *C_i,t_*_1_, …, *C_i,n_*_1_ are recorded, with each target’s detection number recorded as one. The subsequent frame is then utilized as the matching image, denoted as *j* = 2, where all potential targets *C_j,_*_1_, …, *C_j,t_*_2_, …, *C_j,n_*_2_ are identified and their respective parameters recorded. For every potential target in the reference frame, the distance *Dis*(*C_i,t_*_1_, *C_j,t_*_2_), area change rate *Ratio_area_*(*C_i,t_*_1_, *C_j,t_*_2_), and the variation of aspect ratio *Ratio_width&height_*(*C_i,t_*_1_, *C_j,t_*_2_) are calculated in relation to all potential targets in the matching frame. A successful match updates only if the distance, area change rate, and variation of aspect ratio meet the corresponding thresholds; as a result, the position and size information are updated, and detection numbers of *C_i,t_*_1_ are increased by one. The information about these confirmed targets is then fed into the matching process for the subsequent five-frame unit. As the matching progresses, the threshold for match count is elevated, ensuring the continuous identification and output of authentic targets. *Dis*(,), *Ratio_area_*(,), *and Ratio_width&height_*(,) are defined as follows:

(17) $$Dis(C_{i,t1},C_{j,t2})=\sqrt{{\left(x_{i,t1}-x_{j,t2}\right)}^{2}+{\left(y_{i,t1}-y_{j,t2}\right)}^{2}},$$

(18) $$Ratio_{area}(C_{i,t1},C_{j,t2})=\sqrt{{\left(a_{i,t1}-a_{j,t2}\right)}^{2}/\left(a_{i,t1}\times a_{j,t2}\right)},$$

(19) $$Ratio_{width\&height}(C_{i,t1},C_{j,t2})=\sqrt{{\left(R_{i,t1}-R_{j,t2}\right)}^{2}/\left(R_{i,t1}\times R_{j,t2}\right)},$$

where (*x*, *y*) is the upper-left coordinate of the minimum external bounding box and *a* and *R* are the area and aspect ratio of the bounding box, respectively. The threshold of distance *T_dis_* is set to 20 pixels, and the area change threshold *T_Rarea_* and the threshold of variation of aspect ratio *T_Rwh_* are set to 0.5.

## 3. Results

In this section, a series of comparative experiments were performed on infrared image sequences across seven diverse scenarios to test the effectiveness of the proposed method in identifying both bright and dark ships. As for comparison, we chose the several algorithms that are commonly utilized in infrared ship detection tasks, to be specific, a method proficient in detecting dark targets within intricate scenarios [35], as well as two methods capable of simultaneously detecting both bright and dark targets [36,38]. The comparative experiments were executed using MATLAB2018a.

### 3.1. Test Dataset

**Figure 9:**
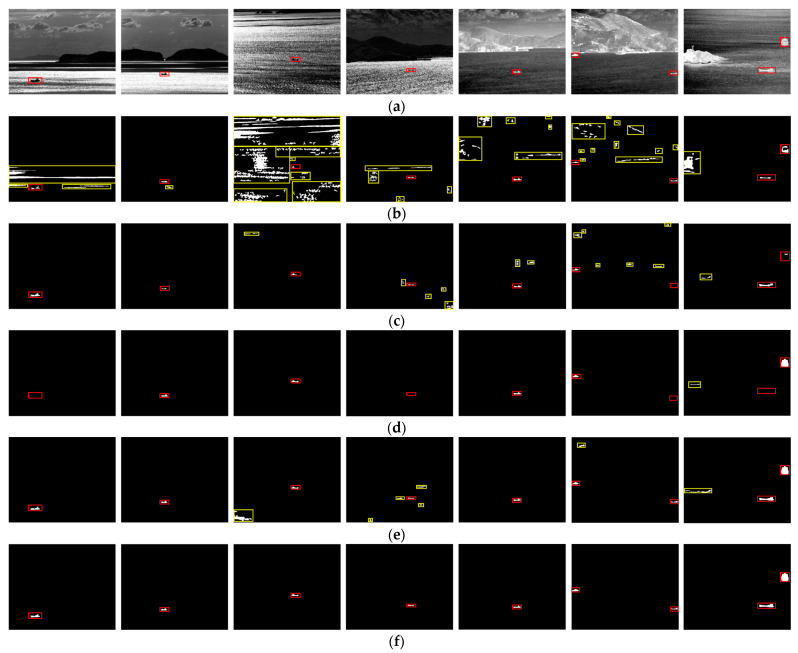
Detection results of different methods. (**a**) The original images of Seq1–Seq9. (**b**) The detection results of the ITDBE. (**c**) The detection results of the MRMF. (**d**) The detection results of the TFMSER. (**e**) The single-frame detection results of the proposed method. (**f**) The multi-frame matching results of the proposed method. The red rectangles represent the positions of targets, and the yellow rectangles represent the false alarm.

The test dataset consists of seven sequences of infrared images, labeled Seq1–7. Each sequence features a resolution of 640 × 512 pixels and contains 1 or 2 bright/dark ships, as depicted in Figure 9a. The sea surface background in sequences Seq2, Seq5, and Seq6 is rather gentle, whereas Seq3 and Seq4 are full of fish scale patterns of varying sizes. There are large-scale dark bands in Seq1–Seq3, and all the sequences contain islands and reefs of diverse shapes. The Table 3 lists the specific details of the seven sequence sets. Notably, a median filter with a 3 × 3 template size was employed in images from Seq3 and Seq4 to achieve preliminary noise reduction. Moreover, taking into account the diversity of ship target size, shape, and background complexity, the Intersection over Union (IOU) threshold has been consistently established at 0.4 to ensure a standardized assessment across all scenarios. In addition to Seq4, the smoothing parameters λ, σ, and the number of iterations were set to 0.015, 3, and 3, respectively. To avoid incorrectly eliminating the narrow ship in Seq4, the σ was set to 1. Apart from this, the step size Δ range of MSER extraction was experimentally set to [3.5, 8].

### 3.2. Qualitative Comparison

In this section, ITDBE [35], which is adept at detecting dark targets within multifaceted scenes, alongside MRMF [36] and TFMSER [38], both capable of discerning light and dark polar targets, was chosen to test the infrared sequences. The ITDBE approach commences with Gaussian differential processing to enhance target contrast, followed by the identification of candidate targets using a multi-scale central difference method inspired by the human visual attention model. The process culminates with adaptive threshold segmentation, with extra grayscale inversion applied to Seq5–7 to align with the algorithmic prerequisite. As shown in Figure 9b, ITDBE demonstrates proficiency in detecting ships when there is obvious contrast with the background and the target’s area is small. However, numerous small but prominent interferences were also inadvertently detected, and they performed worse when dealing with larger targets. Moreover, in more complex scenes, adaptive threshold segmentation struggles to achieve an optimal balance between completely extracting the target and minimizing interference. The MRMF innovatively incorporates grayscale morphological reconstruction into the task of infrared ship detection, which generates a saliency map for potential bright and dark targets by executing dual reconstruction operations on the input image, followed by repeated adaptive threshold segmentation. The results are further refined by considering the shape features and the maximal eigenvalue of the structural tensor for final discernment. As observed in Figure 9c, MRMF generally succeeds in detecting ship targets with pronounced contrast. Nevertheless, it falters when the numerical “salience” of the target is comparable to or less than the interference, leading to missing. The challenge is exacerbated in sequences Seq6 and Seq7, where targets with intermediate grayscale values may be overlooked. The TFMSER leverages the grayscale uniformity and spatiotemporal stability of ships, which begins by extracting MSERs in adjacent frames, then employs spatiotemporal features to extract stable candidate regions while suppressing clutter, and ultimately utilizes shape features to identify the real ships. The results shown in Figure 9d indicate that this method typically incurs the fewest false alarms and exhibits robust detection capabilities for stable targets in simple scenarios, which are determined by the strict spatiotemporal limits. However, detection may fail when the ship is small or set against a complex background; even a relatively stable ship target may experience significant alterations in the extracted MSERs from frame to frame, leading to mismatches and missing. Figure 9e show the single-frame detection results of the proposed method, illustrating its capacity to detect ships across all sequences with the utmost precision.

**Figure 10:**
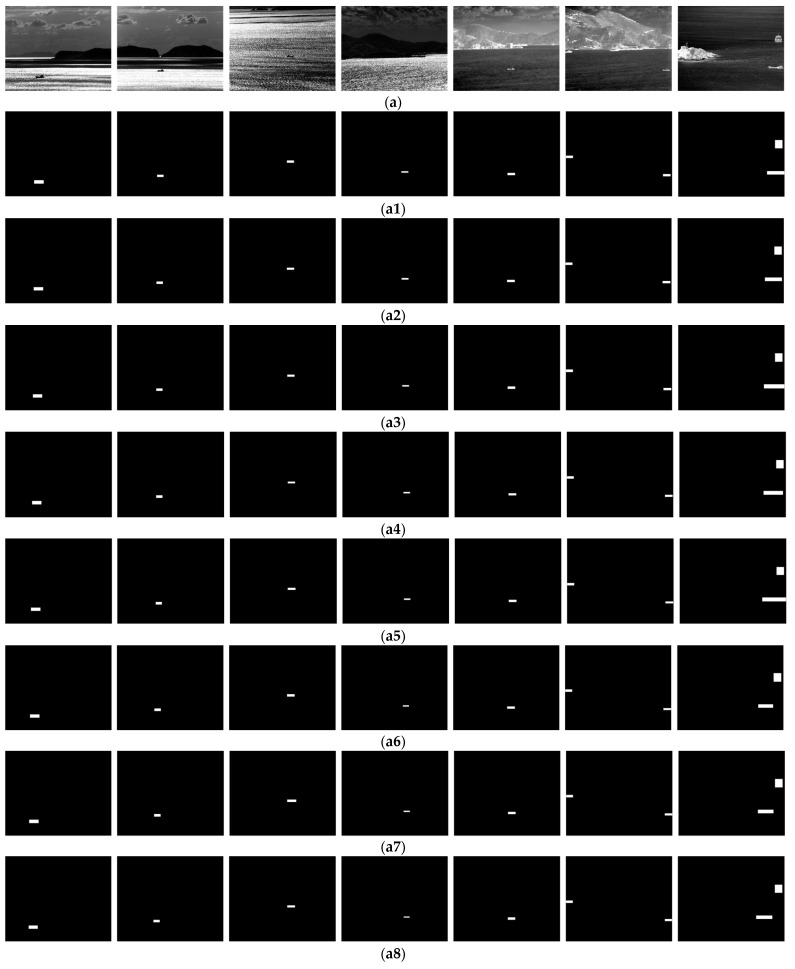

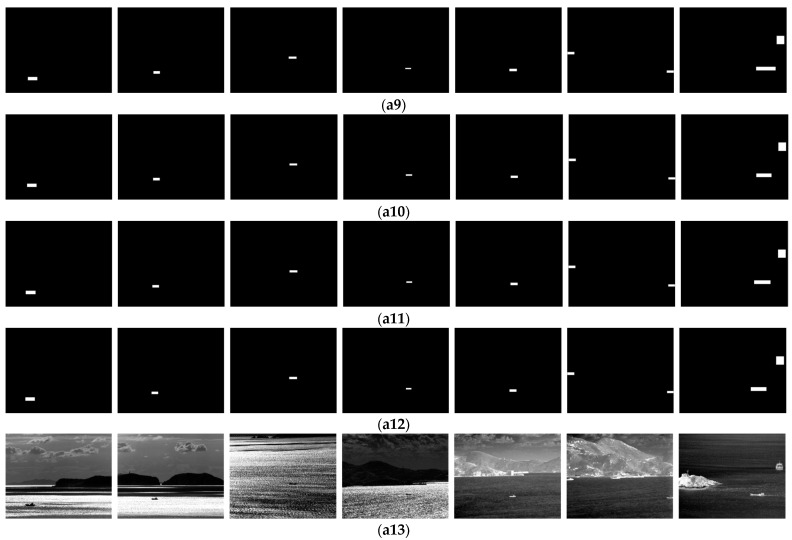
Multi-frame matching result. (**a**) The first frame of each set of sequences. (**a1**–**a12**) are the results of multi-frame matching of the 25th, 50th, …, 25 × *i*th, …, and 300th (*i* = 1, 2, ... , 12) frames of the seven sequences, respectively. (**a13**) The last frame of each set of sequences.

Moreover, it should be noted that, due to the influence of factors such as the performance of the infrared detectors and the complexity of the maritime environment, it is difficult for the existing methods to reasonably infer the potential bright and dark polarity and shape features of the target in the absence of sufficient prior knowledge. In order to ensure the detection rate, current methods (including those discussed in this paper and the proposed method) that simultaneously process bright and dark targets through dual channels often yield a substantial number of false alarms in the channel opposite to the actual target’s polarity. To address this, we augment the single-frame detection result with a multi-frame matching technique to more effectively extract true targets. As illustrated in Figure 10, the integration of the multi-frame matching strategy with the single-frame detection method notably eliminates fluctuation interferences. When evaluated across various sequences, the proposed algorithm demonstrates superior performance in comparison to existing methods.

### 3.3. Quantitative Comparison

To facilitate a more objective assessment of the methods, four key performance metrics for quantitative comparison were employed: the detection rate *D_p_*, false alarm rate *FAR*, misclassification error *ME*, and relative foreground area error *RAE*. Specifically, *D_p_* and *FAR* reflect precision in ship detection and resilience to interference, respectively. *ME* quantifies the proportion of background pixels misclassified as foreground, while *RAE* represents the area-based proximity of the detection results to the real ships. According to the above analysis, the higher the *D_p_*, the stronger the detection ability of the algorithm. Conversely, a lower *FAR* indicates better resistance to interference. Additionally, lower *ME* and *RAE* values denote a more precise and comprehensive segmentation effect of the ship. The four metrics are defined as follows:

(20) $$ME=1-\frac{\left|B_{O}\cap B_{T}\right|+\left|F_{O}\cap F_{T}\right|}{\left|B_{O}\right|+\left|F_{O}\right|},$$

(21) $$RAE=\left\{\begin{array}{c} \frac{A_{O}-A_{T}}{A_{T}},A_{O}>A_{T}\\ \frac{A_{T}-A_{O}}{A_{T}},others \end{array}\right.,$$

(22) $$D_{p}=\frac{TP}{GT},$$

(23) $$FAR=\frac{FP}{FP+TP},$$

where *B_O_* and *F_O_* denote the number of pixels of the background and the number of pixels of the ship target in the ground truth images, respectively. *B_T_* and *F_T_* represent the number of pixels of the background and the ship target in the detection results, respectively. *A_T_* and *A_O_* are the areas of the ship determined by the ground truths and the detection results, respectively. *TP* quantifies the numbers where a ship target is accurately detected, while *FP* includes the occurrences where non-target elements are identified as ships, and *GT* indicates the total number of real ships that have been manually annotated.

**Figure 11:**
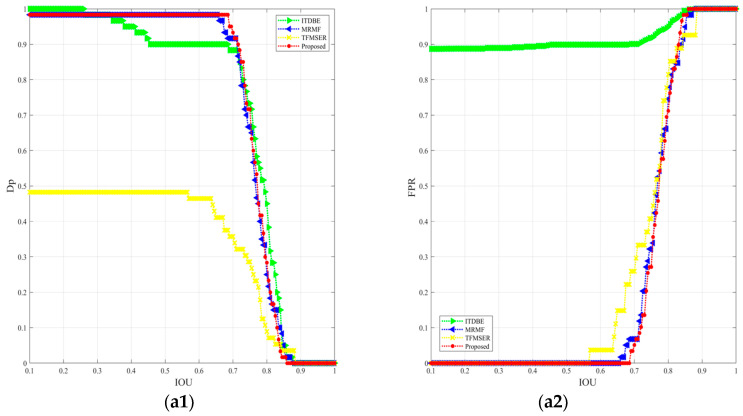

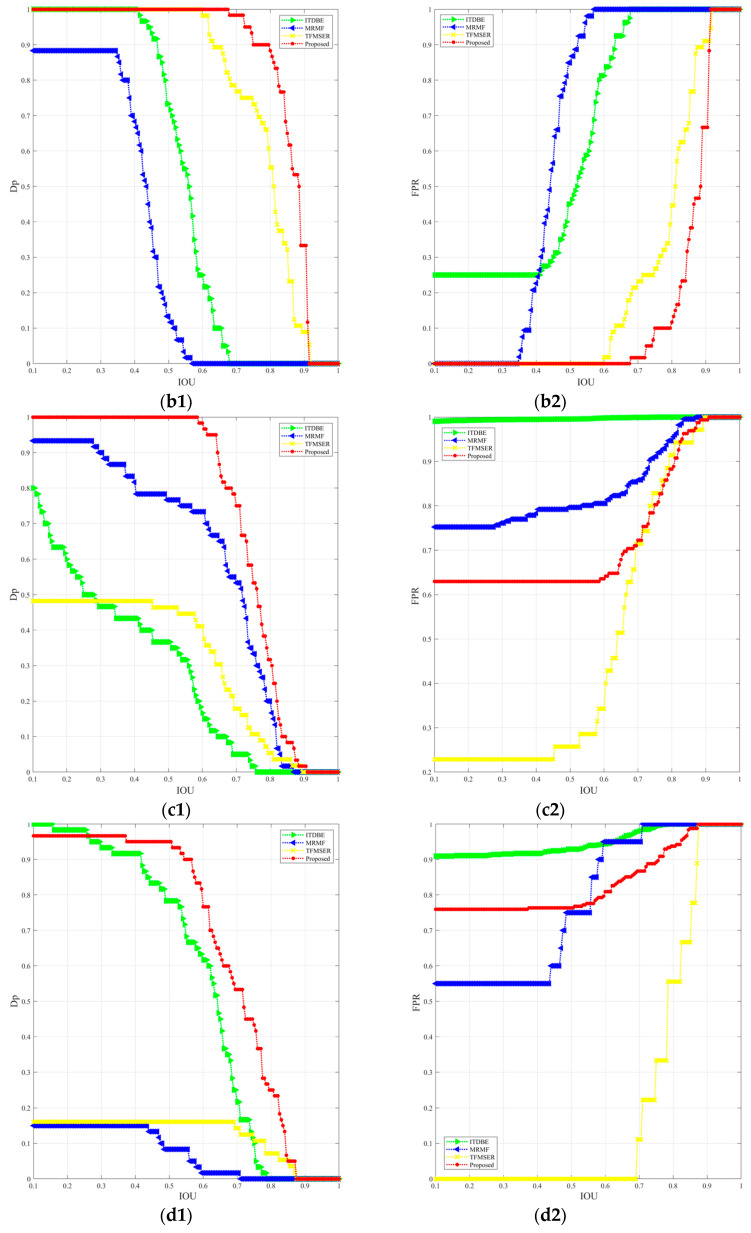

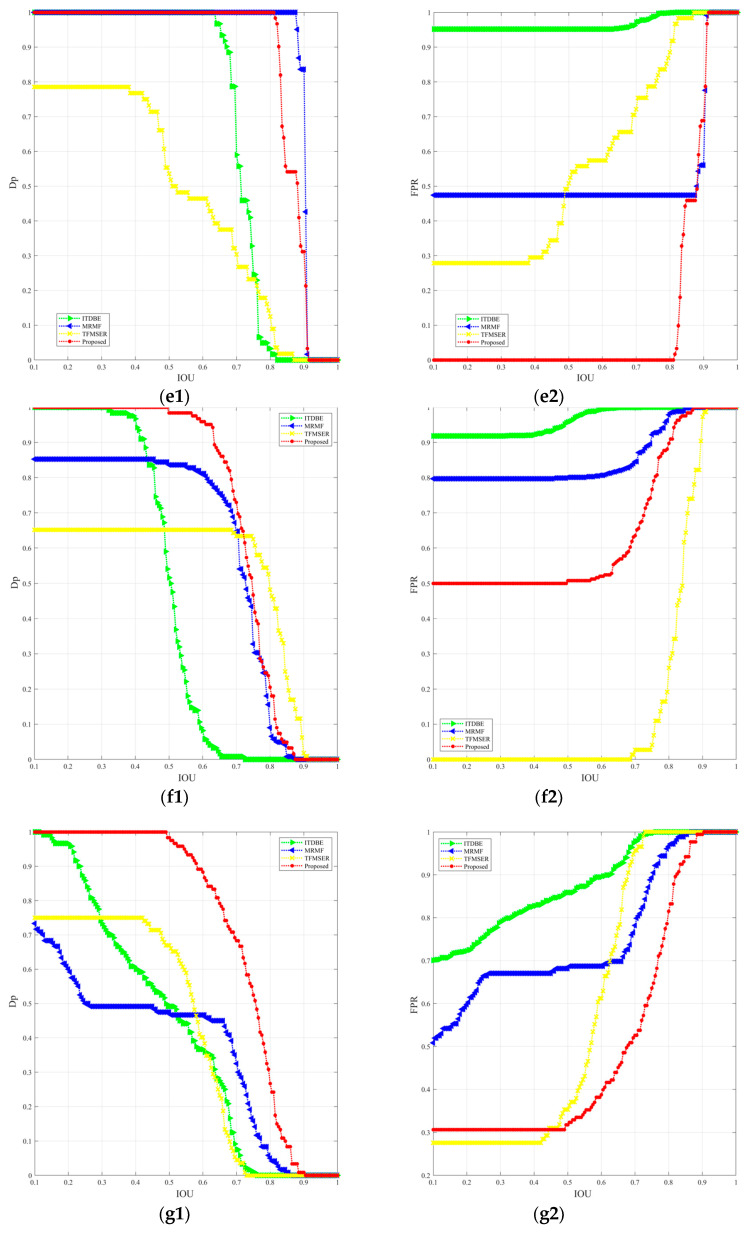
The ROC curves of 7 sequences, with IOUs ranging from 0.1 to 1. (**a1**–**g1**) and (**a2**–**g2**) are the curves of *D_p_* and *FAR* for selected methods in 7 sequences, respectively. The green triangle, blue triangle, yellow “x”, and red dots in the figures denote the results of ITDBE, MRMF, TFMSER, and the proposed single-frame detection method, respectively.

The results of the metrics of the selected methods are shown in the following Table 4, Table 5, Table 6 and Table 7. The results reveal that with the IOU set to 0.4, the proposed method outperforms others in *D_p_*, and the *ME* and *RAE* indicate that the proposed method can extract the ships accurately and completely across most scenarios while maintaining a relatively reasonable *FAR*. Additionally, although the TFMSER maintains the lowest *FAR* in all scenarios, its strict inter-frame matching mechanism also seriously curtails its detection capability in complex scenarios. In contrast, the ITDBE exhibits relatively robust detection ability but is prone to generating an excessive number of false alarms that far exceed the number of targets detected. In complex scenarios, adaptive threshold segmentation may struggle to distinguish between “significant” interference and real targets, resulting in the limited detection and anti-interference capabilities of MRMF in complex scenes such as Seq3–4. Figure 11 exhibits the ROC curves of the selected methods in seven sequences. It can be seen that the ITDBE method shows excellent detection performance in scenarios with simple backgrounds and small ship sizes, while in certain situations, such as Seq1 and Seq4, the entire ship may be detected partially, resulting in a fairly high *D_p_* at low IOU levels, which demonstrates that this method lacks the ability to suppress minor background clutter. The MRMF falls short in detecting small ships and those with intermediate grayscale values. Owing to the strict spatiotemporal feature constraints, the TFMSER exhibits the strongest interference suppression ability, but in turn, this also constrains its detection performances at the edge of the image (such as Seq6 and Seq7) and small ships in complex scenarios. The proposed single-frame detection method showcases the best detection performance in all scenarios. Despite this, the processing method based on dual types of grayscale reconstruction and RTV model smoothing also contributes to the increase in *FAR*. Nevertheless, as previously discussed, a spatiotemporal feature-based multi-frame matching strategy can effectively mitigate fluctuating false alarms.

## 4. Conclusions

In this paper, we introduce a novel infrared image smoothing technique composed of GMR and RTV. Additionally, a detection method considering the grayscale uniformity of ships and integrating shape and spatiotemporal features is established for detecting bright and dark ships in complex maritime scenarios. Initially, the input infrared images undergo *OGMR*(*CGMR*) to preserve dark (bright) blobs with the opposite suppressed, followed by smoothing the image with the RTV to reduce clutter and enhance the contrast of the ship. Subsequently, Maximally Stable Extremal Regions (MSERs) are extracted from the smoothed image as candidate targets, and the results from the bright and dark channels are merged. Shape features are then utilized to eliminate clutter interference, yielding single-frame detection results. Finally, utilizing the spatiotemporal stability of ships and the fluctuation of clutter, true targets are identified through a multi-frame matching strategy. Experimental results demonstrate that the proposed method outperforms ITDBE, MRMF, and TFMSER in seven image sequences, achieving accurate and effective detection of bright and dark polarity ship targets. Our method avoids the use of adaptive threshold segmentation, which may struggle in complex maritime scenes. Instead, the RTV method is introduced into the preprocessing process of infrared images to enhance the suppression effect of fish scale plates, improve the detection effect of ships, and combine underutilized features such as gray uniformity and spatiotemporal stability, hoping to provide new ideas for infrared ship detection tasks.

Despite the excellent detection results of the proposed method, there are still some shortcomings. Primarily, the proposed method is aimed at ships with distinct polarities—bright and dark—and may exhibit unsatisfactory preformation when encountering ships with an uneven grayscale distribution, potentially leading to incomplete or missed detections. Future amendments could incorporate methods like watershed segmentation [56] and region growth [57] to refine the detection of unevenly distributed ships. Additionally, the proposed method avoids the use of adaptive threshold segmentation due to its limited adaptability in complex scenarios. A simple and effective evaluation mechanism for describing the scenarios and adjusting the parameters of the methods still remains necessary. For example, an evaluation method based on the statistical results of the image blocks in [18] could adaptively guide mean drift filters. Therefore, we will also focus on the evaluation mechanism of the scenario as a key direction for future studies. Lastly, with the rapid advancements in deep learning, there is an aspiration to integrate deep learning-based methods into infrared ship target detection tasks. This may involve using deep learning to refine and summarize the shape features of targets, potentially replacing traditional manual designed features to achieve more effective and robust ship detection. Furthermore, leveraging deep learning methods to explore image features at a deeper and more abstract level enables the extraction of richer semantic information, which can facilitate distinguishing different components within complex maritime scenarios (such as separating sea surfaces, islands, and the sky) and constructing image models and field data that can accurately describe such scenarios. These insights may provide novel ideas for achieving more effective and robust detection.

## Figures and Tables

**Table 1:** Quantitative evaluation of the proposed smoothing method.

Sequences	*PSNR* (dB)	*mSSIM*
Seq1	20.7134	0.7257
Seq2	22.2995	0.7258
Seq3	18.9157	0.3616
Seq4	20.0236	0.6681
Seq5	22.5759	0.6064
Seq6	20.4198	0.5993
Seq7	24.0667	0.4799

**Table 2:** Ranges of shape features.

Feature	Minimum	Maximum
*Ratio_height&width_*	1.6	11
*Compactness*	12.9445	317.1574
*Rectangularity*	0.3008	0.9788
*Ratio_up&down_*	<1	
*Area*	81	1500

**Table 3:** Details of the test sequences.

Sequences	Frames	Target	Background
Seq1	300	1 dark ship moving to the left	Complex, with a large-scale dark band
Seq2	300	1 dark ship moving to the left	Rather gentle, with a large-scale dark band
Seq3	300	1 stationary dark ship	Rather complex, with a dark band and islands
Seq4	300	1 dark ship moving to the right	Complex, with a dark band and small islands
Seq5	300	1 stationary bright ship	Gentle, with artificial buildings and a mountain
Seq6	300	2 stationary bright ships	Gentle, with artificial buildings and a mountain
Seq7	300	2 bright targets: one stationary and one moving to the left	Rather complex, with artificial buildings, an island, and reefs

**Table 4:** Average *Dp* of the selected methods on the test dataset. The bold emphasis represents the best results.

Sequence	ITDBE	MRMF	TFMSER	Proposed
Seq1	0.9500	**0.9833**	0.4643	**0.9833**
Seq2	**1.0000**	0.6833	**1.0000**	**1.0000**
Seq3	0.4333	0.8167	0.4821	**1.0000**
Seq4	0.9167	0.1500	0.1607	**0.9500**
Seq5	**1.0000**	**1.0000**	0.7857	**1.0000**
Seq6	0.9672	0.8524	0.6518	**1.0000**
Seq7	0.6000	0.4916	0.7500	**1.0000**

**Table 5:** Average *FAR* of the selected methods on the test dataset. The bold emphasis represents the best results.

Sequence	ITDBE	MRMF	TFMSER	Proposed
Seq1	0.8933	**0.0000**	**0.0000**	**0.0000**
Seq2	0.2500	0.2264	**0.0000**	**0.0000**
Seq3	0.9948	0.7832	**0.0357**	0.6296
Seq4	0.9171	0.5500	**0.0000**	0.7635
Seq5	0.9518	0.4741	0.2787	**0.0000**
Seq6	0.9210	0.7969	**0.0000**	0.5000
Seq7	0.8278	0.6704	**0.2075**	0.3064

**Table 6:** Average *ME* of the selected methods on the test dataset. The bold emphasis represents the best results.

Sequence	ITDBE	MRMF	TFMSER	Proposed
Seq1	0.1687	**0.1240**	0.6114	**0.1812**
Seq2	0.3962	0.6087	**0.0855**	0.0910
Seq3	0.1199	0.3405	0.5944	**0.1575**
Seq4	**0.1226**	0.8994	0.8426	0.2176
Seq5	0.1277	0.0540	0.2446	**0.0402**
Seq6	0.4869	0.3684	0.4984	**0.2466**
Seq7	0.4730	0.5912	0.3252	**0.1276**

**Table 7:** Average *RAE* of the selected methods on the test dataset. The bold emphasis represents the best results.

Sequence	ITDBE	MRMF	TFMSER	Proposed
Seq1	0.9335	0.1575	0.6114	**0.1487**
Seq2	0.2436	0.6690	0.0978	**0.0977**
Seq3	0.9939	**0.3739**	0.5560	0.4281
Seq4	0.9342	0.7863	0.8940	**0.6206**
Seq5	0.9496	0.2454	0.3726	**0.2011**
Seq6	0.8402	0.4964	0.3502	**0.3056**
Seq7	0.9342	0.7863	0.9207	**0.5939**

## Data Availability

The data that support the findings of this study are available from the corresponding author upon reasonable request.
